# Comparative Analysis of Global Gene Expression and Complement Components Levels in Umbilical Cord Blood from Preterm and Term Neonates: Implications for Significant Downregulation of Immune Response Pathways related to Prematurity

**DOI:** 10.7150/ijms.46339

**Published:** 2020-07-11

**Authors:** Dorota Gródecka-Szwajkiewicz, Zofia Ulańczyk, Edyta Zagrodnik, Karolina Łuczkowska, Dorota Rogińska, Miłosz P. Kawa, Iwona Stecewicz, Krzysztof Safranow, Przemysław Ustianowski, Sławomir Szymański, Bogusław Machaliński

**Affiliations:** 1 Department of General Pathology, Pomeranian Medical University in Szczecin, Szczecin, Poland; 2 Department of Biochemistry and Medical Chemistry, Pomeranian Medical University in Szczecin, Szczecin, Poland; 3 Department of Perinatology, Obstetrics and Gynecology, Pomeranian Medical University in Szczecin, Szczecin, Poland; 4 Department of Obstetrics and Pathology of Pregnancy, Pomeranian Medical University in Szczecin, Szczecin, Poland

**Keywords:** premature birth, complement system proteins, immunity, gene expression

## Abstract

**Background:** Preterm birth is the most frequent cause of neonatal death, but its aetiology remains unclear. It has been suggested that the imbalance of immunological mechanisms responsible for maintaining pregnancy is contributing to preterm birth pathogenesis. We aimed to investigate global gene expression and the levels of several complement system components in umbilical cord blood samples from preterm neonates and compare them to term newborns. We sought to examine how differentially expressed genes could affect various immune-related pathways that are believed to be crucial factors in preterm birth.

**Material and methods:** We enrolled 27 preterm infants (<37 weeks GA) and 52 term infants (>37 weeks GA), from which umbilical cord blood samples were collected. From these samples, peripheral blood mononuclear cells were isolated and subsequent RNA isolation was performed. We used Affymetrix Human Gene 2.1 ST Array Strip for microarray experiment and DAVID resources for bioinformatics analysis of the obtained data. Concentrations of C2, C3a, C5/C5a, C9, FactorD, Properdin were measured in umbilical cord blood plasma samples using multiplex fluorescent bead-based immunoassays using Luminex technology.

**Results:** The levels of C3a and C5/5a were significantly elevated in preterm neonates compared to term babies, whereas C9 concentration was evidently increased in term infants. The expression of 250 genes was upregulated at least 2-fold and 3781 genes were downregulated at least 2-fold in preterm neonates in comparison with term infants. Functional annotation analysis revealed that in preterm infants in comparison to term babies there was a significant downregulation of genes encoding several Toll-like receptors, interleukins and genes involved in major signalling pathways (e.g. NF-κB, MAPK, TNF, Notch, JAK) and vital cellular processes (e.g. intracellular signal transduction, protein ubiquitination, protein transport, RNA splicing, DNA-templated transcription).

**Conclusions:** Preterm birth results in immediate and long-term complications. Our results indicate that infants born prematurely show significant differences in complement components concentration and a downregulation of over 3,000 genes, involved mainly in various immune-related pathways, including innate immune response, phagocytosis and TLR function, when compared to full-term babies. Further studies on larger cohorts are needed to elucidate the role of immunity in prematurity.

## Introduction

Preterm birth (PTB), defined by the World Health Organization (WHO) as a delivery prior to 37 completed weeks of gestation, accounts for roughly 5-18% of all births worldwide 1. Although PTB rates vary across the globe, one thing is common: PTB remains the most frequent cause of neonatal death 2. Even if a preterm infant survives, prematurity-related complications are tremendous and include neonatal respiratory conditions, necrotizing enterocolitis, sepsis, neurological conditions, visual and hearing problems 3. The higher rates of long-term morbidity in prematurely born babies in comparison to term newborns are attributed to immaturity of preterm infant's organs. In fact, gestational age (GA) at delivery and the risk of neonatal morbidity and mortality are inversely related with each other 4. PTB has also been linked with complications later in life, e.g. higher rates of hospital admissions, poorer neurodevelopmental outcomes, behavioural and social difficulties 5. The medical impact and socio-economic burden of preterm birth consequences make better understanding and therefore possible prevention of PTB a public health priority.

However, PTB aetiology remains not fully understood. Despite the multitude of risk factors identified 6, the majority of preterm births do not have a specific risk factor. Several different pathological processes have been implicated in preterm birth, including ischemia, stress, infections, cervical diseases and other 7. One particular factor that has been increasingly implicated in PTB pathogenesis is dysregulation of a fine balance of immunological mechanisms normally responsible for maintaining pregnancy 8. The maternal immune system faces a considerable challenge during pregnancy, as it has to tolerate the paternal alloantigens expressed in foetal tissues and simultaneously maintain effective protection against microbes with innate and adaptive immunity mechanisms 9.

According to Gomez-Lopez et al., during late pregnancy, the pro-inflammatory nuclear factor-kB (NF-κB) signalling pathway is activated and that leads to recruitment of circulating maternal leukocytes at maternal/foetal interface by chemotaxis 10. Premature shift from anti-inflammatory to this pro-inflammatory state is believed to contribute to a loss in foetal-maternal tolerance and consequent labour initiation, leading to preterm delivery 11. The imbalance of both innate and adaptive immunity systems components have been implicated in PTB or miscarriage, but the role of immunity deficiencies in preterm infants seems to be even more profound 12. Numerous studies have shown that immune system of preterm neonates is more immature than that of term ones 13, 14. In fact, both innate and adaptive immunity of preterm babies is diminished, as are the interactions between these two systems. It has been also hypothesised that PTB may shape preterm infants' immune system via various common pre- and postnatal events associated with it that have the potential to influence immunity 15.

Neonates are forced to rely mainly on their innate immune defence, because their inexperienced adaptive immune system fully develops only later, in the early childhood 16. Preterm infants are more prone to bacterial infections than babies born at term and there are several reasons for that, including prolonged intensive care, but the immaturity of innate immune system remains a major cause 13. Term infants could be considered 'lucky' to have received maternal antibodies that serve as supplemental protection, whereas preterm infants 'missed' the largest part of transfer of maternal antibodies, as it increases with foetal age 17. As one of the most important constituents of innate immunity, the role of complement in PTB and prematurity has also been implicated 18. The maturity of complement components and their concentrations seems inadequate even in healthy term newborns, and these deficiencies are even more profound in preterm neonates 18. Complement activation is crucial in pathogen opsonization, anaphylatoxins synthesis, leukocyte recruitment and bacteria lysis 19. However, in preterm infants, complement activation pathways (classical, alternative, lectin) show profound dysregulation in their pathogen-killing abilities 20. Although the role of immunity in prematurity is getting more attention, its exact role in this condition and how differentially expressed genes could affect various immune-related pathways leading to PTB and/or affecting preterm newborns remains not fully understood.

In our previous studies, we have investigated umbilical cord blood (UCB) expression levels of small non-coding RNAs (miRNAs) potentially involved in angiogenesis control in preterm newborns and term neonates 21. In this study, we aimed to investigate global gene expression in UCB samples from preterm neonates and compare it to term newborns. In addition, we sought to examine how these differentially expressed genes could affect various immune-related pathways that are said to be contributing to preterm birth. Moreover, we aimed to compare the levels of several crucial components of complement system in UCB plasma samples between preterm and term infants.

## Methods

### Study groups characteristics

In total, 27 preterm infants born <37 weeks GA and 52 term infants born at >37 weeks GA all appropriate-for-gestational-age were enrolled in this study from the Department of Obstetrics and Gynaecology of the Pomeranian Medical University in Szczecin, Poland. We excluded subjects with severe congenital malformations, known chromosomal abnormalities, intracranial haemorrhage, cyanotic heart defects, inherited metabolic disorders, severe anaemia, congenital infections and severe infectious diseases, maternal history of tobacco and/or alcohol abuse; maternal infections were also excluded. For each recruited neonate we documented sex, GA, birth weight and physical measurements, Apgar score, clinical course. The study adhered to the tenets of the Declaration of Helsinki, and approval was obtained from the Local Ethics Committee. All parents provided written informed consent for their children participation in the study.

### Umbilical cord blood collection

We collected autologous umbilical cord blood from all patients at birth in accordance with NetCord-FACT International Standards for Cord Blood Collection 22. Collections were performed in utero as described previously 23, using a collection bag system with citrate-phosphate-dextrose (CPD) as anticoagulant. UCB samples were then centrifuged (2000 rpm, 4°C, 10 min), and the plasma was removed and stored (-20°C to -80°C) until further assayed. Next, we used BD Pharm Lyse lysing buffer (BD Biosciences, San Jose, CA, United States) for red blood cells lysis to obtain peripheral blood mononuclear cells (PBMCs).

### Luminex assay

Concentrations of C2, C3a, C5/C5a, C9, FactorD, Properdin were measured in UCB plasma using multiplex fluorescent bead-based immunoassays (Luminex Corporation, Austin, TX, USA). The procedure was performed according to the manufacturer's protocol, as previously described 24. In brief, 50 μL of blank, standards and samples were added to the plate together with Microparticle Cocktail. This was followed by incubation step for 2 hours in the dark at room temperature on horizontal orbital microplate shaker (800rpm). Then, 100 µL of wash buffer was used to was the wells three times. Next, 50 µL of biotin-antibody cocktail was added to the plate and incubated for 1 hour in the dark. After subsequent washing step, Streptavidin-PE was added (50 µL) to each well and incubated in the dark for 30. After washing, the microspheres in each well were resuspended in wash buffer (100 µL) and shaken for 2 minutes. Finally, the plate was read on the Luminex 200 analyzer. The tested proteins concentrations were calculated from seven different standard curves that present median fluorescence intensity vs protein concentration.

### RNA isolation

The mirVana™ miRNA Isolation Kit (Thermo Fisher, Waltham, MA, USA) was used for total RNA isolation from collected umbilical cord blood cells (1x10^6^). Concentration and quality of the obtained RNA was assessed using Epoch spectrophotometer (Biotek, Winooski, VT, USA).

### Affymetrix GeneChip Microarray and Data Analysis

Total RNA isolated from UCB samples was pooled to generate one sample per group for microarray experiment. The procedure was performed according to the manufacturer's protocol, as previously described 21. Generation of sense strand cDNA from the total RNA and subsequent fragmentation and labelling steps were made using GeneChip™ WT PLUS Reagent Kit (Thermo Fisher Scientific, Waltham, MA, USA). Finally, the sample was hybridized onto an Affymetrix Human Gene 2.1 ST Array Strip. Affymetrix GeneAtlas System was used for hybridization, fluidics and scanning steps. Subsequent analyses were performed using BioConductor software. We used Robust Multiarray Average (RMA) normalisation algorithm (from “Affy” library) for normalisation, background correction, and calculation of the expression levels of examined genes.

### DAVID

For functional annotation and enrichment analysis, we used DAVID Bioinformatics Resources (Database for Annotation, Visualization, and Integrated Discovery) at http://david.abcc.ncifcrf.gov, as previously described 25, 26. Functional annotation charts generated by DAVID with overrepresented gene annotations are shown as bubble plots from BACA BioConductor package (https://cran.r-project.org/web/packages/BACA/BACA.pdf). The following criteria were applied to generate bubble plots: *p-*value < 0.5, adjusted method = Benjamini 27, and minimal number of genes per group = 5. Groups of genes that meet this criteria are shown in a graph, where the bubble size is indicative of the number of genes represented in the corresponding annotation and their down- or up-regulation.

### Statistical analysis

The nonparametric Mann-Whitney test was used to compare values between study groups, because in majority of cases the quantitative variables distribution notably differed from the normal distribution. Spearman's rank correlation coefficient (Rs) was used to measure the strength of associations between gestational age and concentrations of each tested complement component. The sign of the Rs value indicates the direction of the association (positive or negative), while higher absolute value (closer to -1 or +1) indicates stronger association. We considered p<0.05 statistically significant. Statistica 13 software (Dell Inc., OK, USA) was used for statistical analysis.

## Results

### Study Groups Characteristics

In total, 27 preterm and 52 term neonates were enrolled in this study. The characteristics of the study groups are summarized in Table 1. The preterm neonates differed from term neonates in most of the recorded measurements, including Apgar scores, arterial-blood gas test results, blood cell counts and physical measurements. The groups did not differ in CRP and IL-6 concentrations, indicating lack of apparent acute inflammation and potential pro-inflammatory status of both groups.

### UCB plasma complement component levels

**Figure 1:**
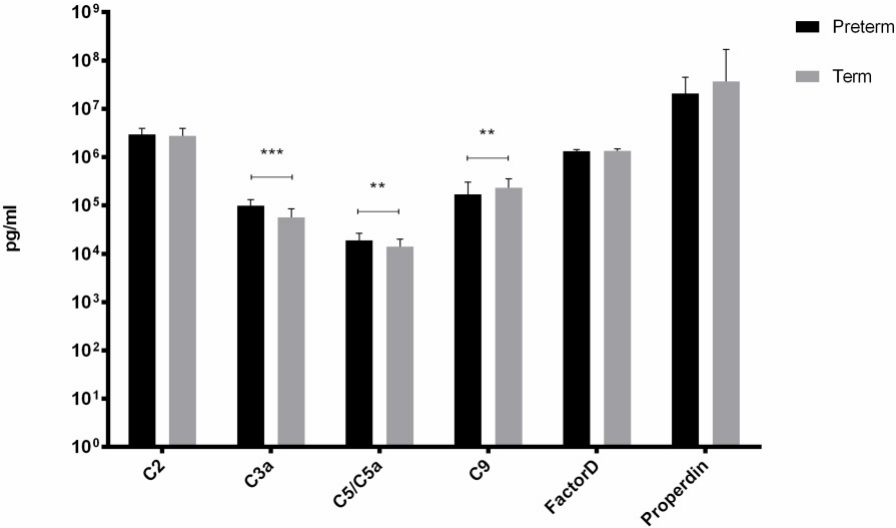
Complement components levels in UCB from preterm and term infants (**p-value <0.01;*** p-value< 0.001).

From six factors tested, we observed differences in concentration of C3a, C5/C5a and C9 between the study groups (Figure 1). The mean concentrations of C3a and C5/5a were significantly elevated in preterm neonates comparing to term babies (p<0.001 and p<0.01, respectively). In contrast, C9 concentration was evidently increased (p<0.01) in term infants when compared with preterm neonates. The rest of the tested factors concentrations did not differ between preterm and term infants.

There was a significant positive correlation between GA and C5/C5a (Rs= +0.35, p=0.01) and C9 (Rs=+0.45, p<0.001) in term infants and between GA and C2 (Rs=+0.45, p=0.02) in preterm infants.

### Gene expression profile in PBMCs isolated from UCB samples

**Figure 2:**
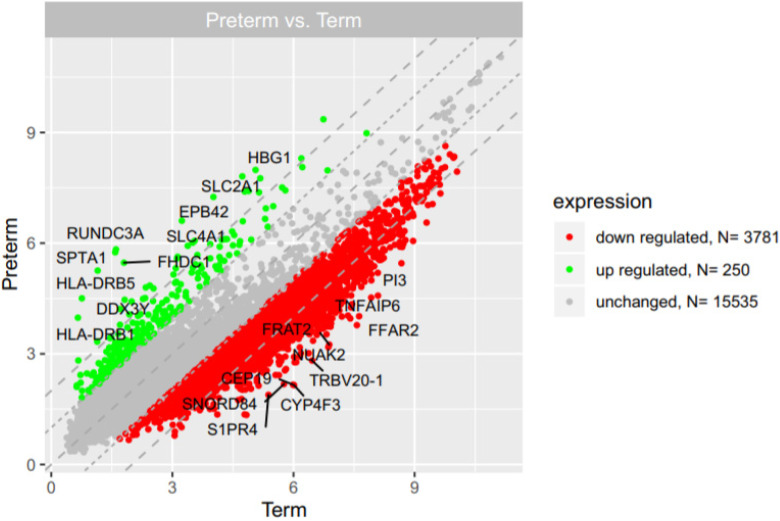
The scatter plot of global gene expression in UCB from preterm neonates when compared to term ones. Red points correspond to downregulated genes (at least 2-fold change, p<0.05), green points show upregulated genes (at least 2-fold change, p<0.05). The graph also contains names of the genes with the highest change in expression.

Microarray analysis showed that expression of 250 genes was upregulated at least 2-fold (fold 18.73 to 2) and 3781 genes were downregulated at least 2-fold (fold -14.55 to -2) in PBMCs isolated from UCB samples from preterm neonates in comparison with term infants (Figure 2). The list of 10 genes with the most significantly up- and downregulated expression is presented in Table 2 and Table 3, respectively.

**Figure 3:**
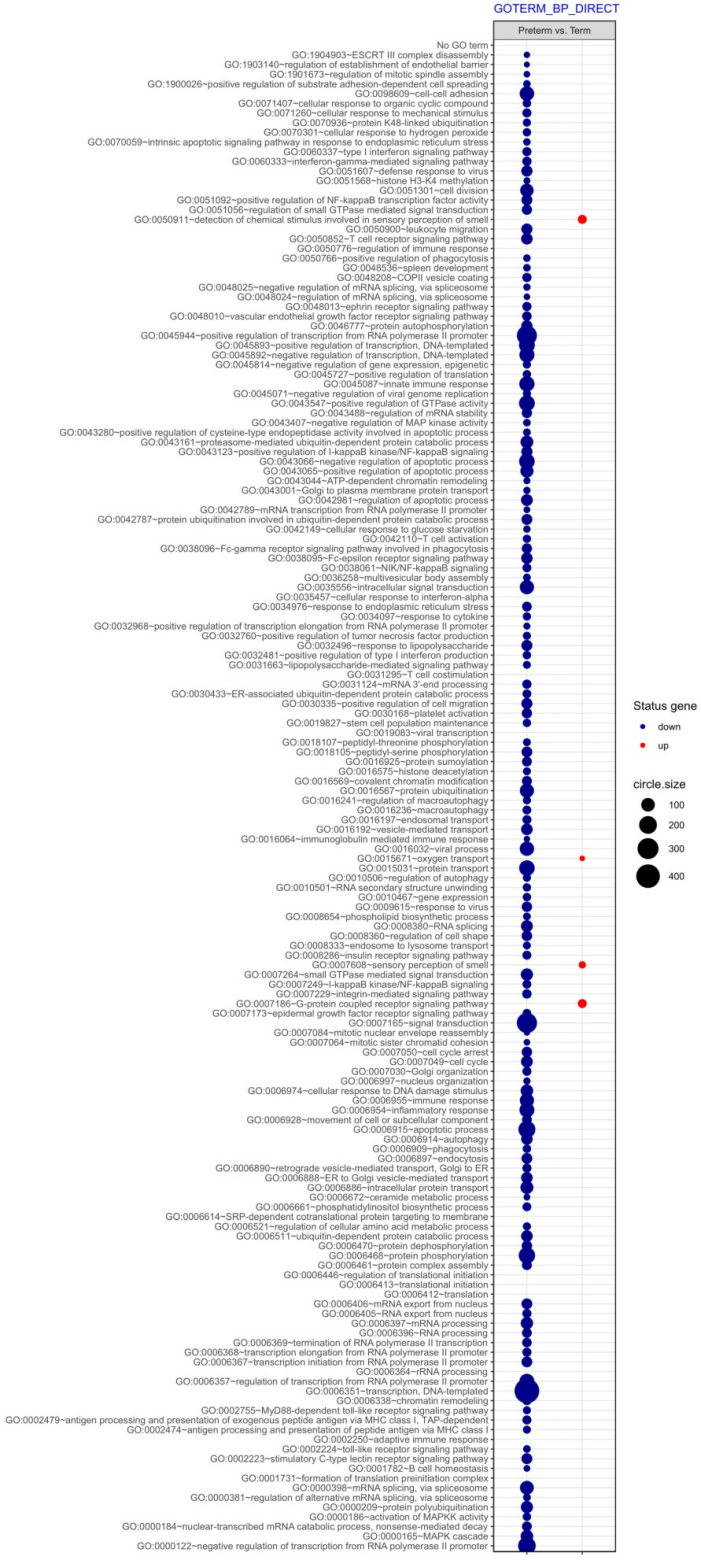
The bubble plot of overrepresented biological processes assigned according to Gene Ontology (GO) classification in PBMCs isolated from UCB samples from preterm neonates in comparison with term infants. Following criteria were applied to assign genes included in the graph to individual processes: adjusted p<0.05, method = Benjamini, minimum number of genes per group = 5. The size of each bubble indicates the number of genes represented in the corresponding annotation and the colour reflects the status of genes in terms of their up- or downregulation.

**Figure 4:**
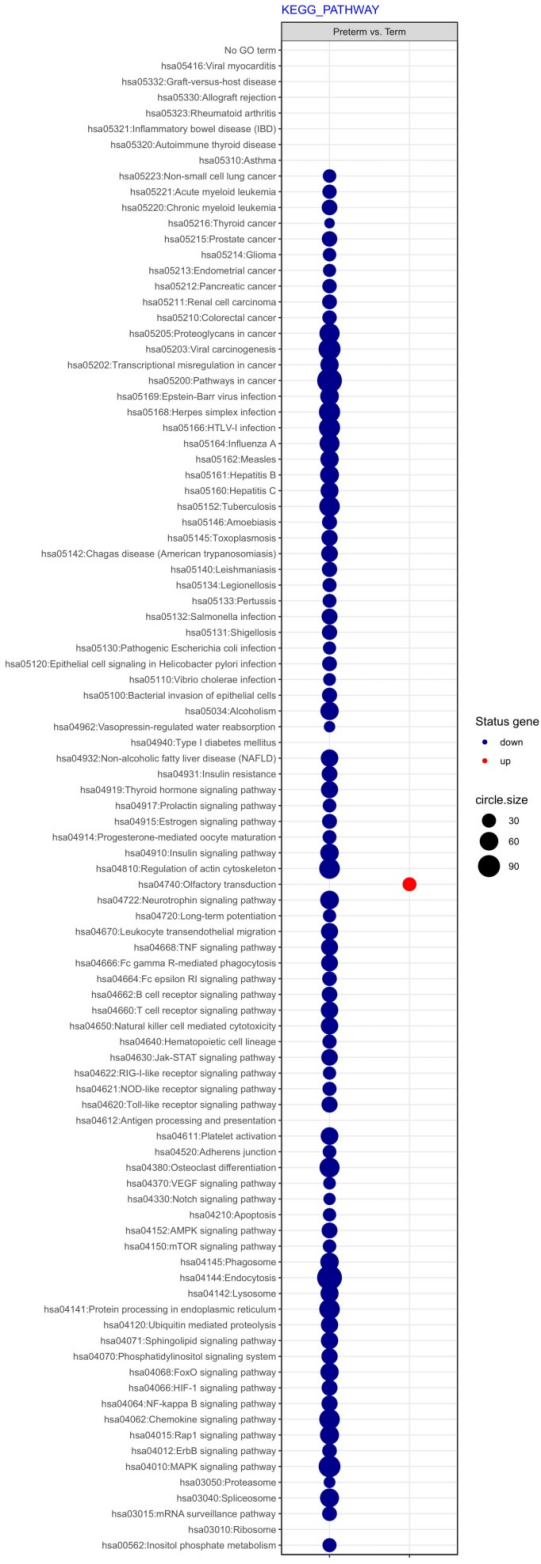
The bubble plot of changed pathways assigned according to KEGG Pathway Database in PBMCs isolated from UCB samples from preterm neonates in comparison with term infants. Following criteria were applied to assign genes included in the graph to individual processes: adjusted p<0.05, method = Benjamini, minimum number of genes per group = 5. The bubble size reflects the number of genes represented in the corresponding annotation and the colour reflects the status of genes in terms of their up- or downregulation.

Next, differentially expressed genes were classified according to the Gene Ontology (GO) Classification of biological processes and KEGG Database of pathways. Analysis of functional annotations mainly identified downregulated processes (e.g. signal transduction, DNA transcription, innate immune response) in PBMCs from UCB of preterm neonates when compared with term infants. The results of these analyses are displayed as separate bubble graphs for biological processes and pathways (Figure 3 and 4, respectively).

**Figure 5:**
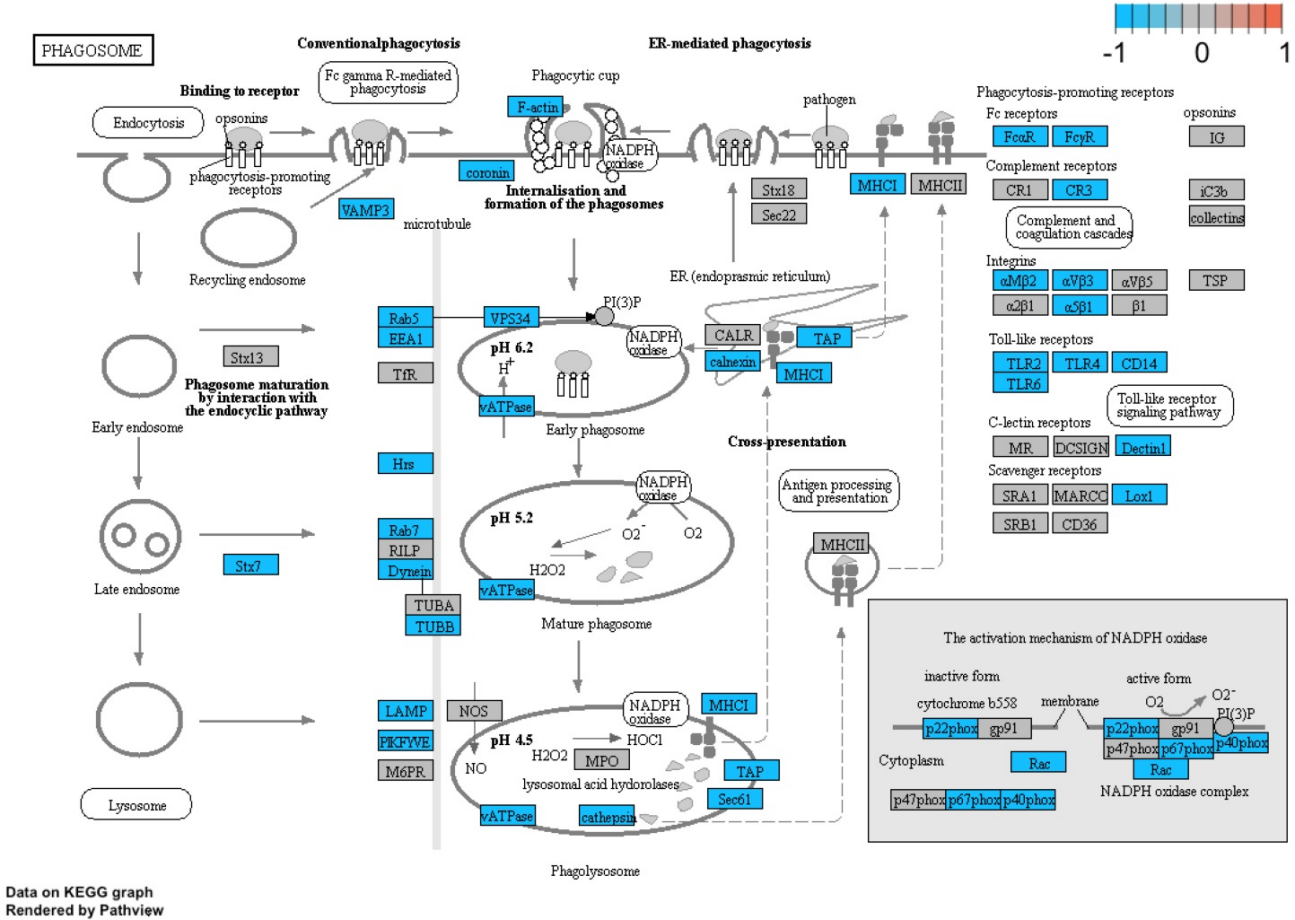
Genes and processes involved in the 04145.Phagosome pathway. The colour scale corresponds to log(FC) value, where FC stands for gene expression fold change when comparing samples from preterm to term neonates.

**Figure 6:**
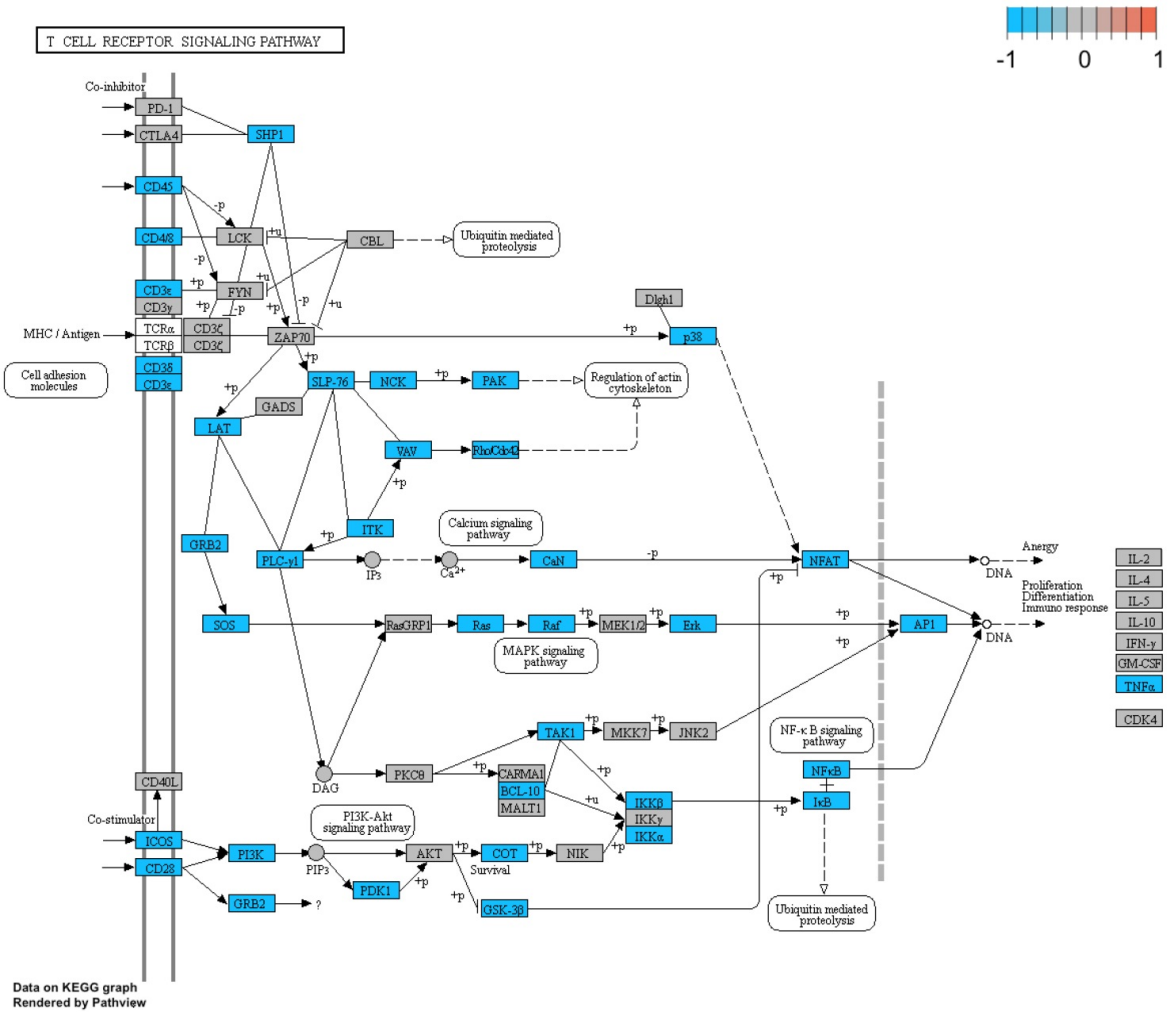
Genes and processes involved in the 04660.T cell receptor signalling pathway. The colour scale corresponds to log(FC) value, where FC stands for gene expression fold change when comparing samples from preterm to term neonates.

**Figure 7:**
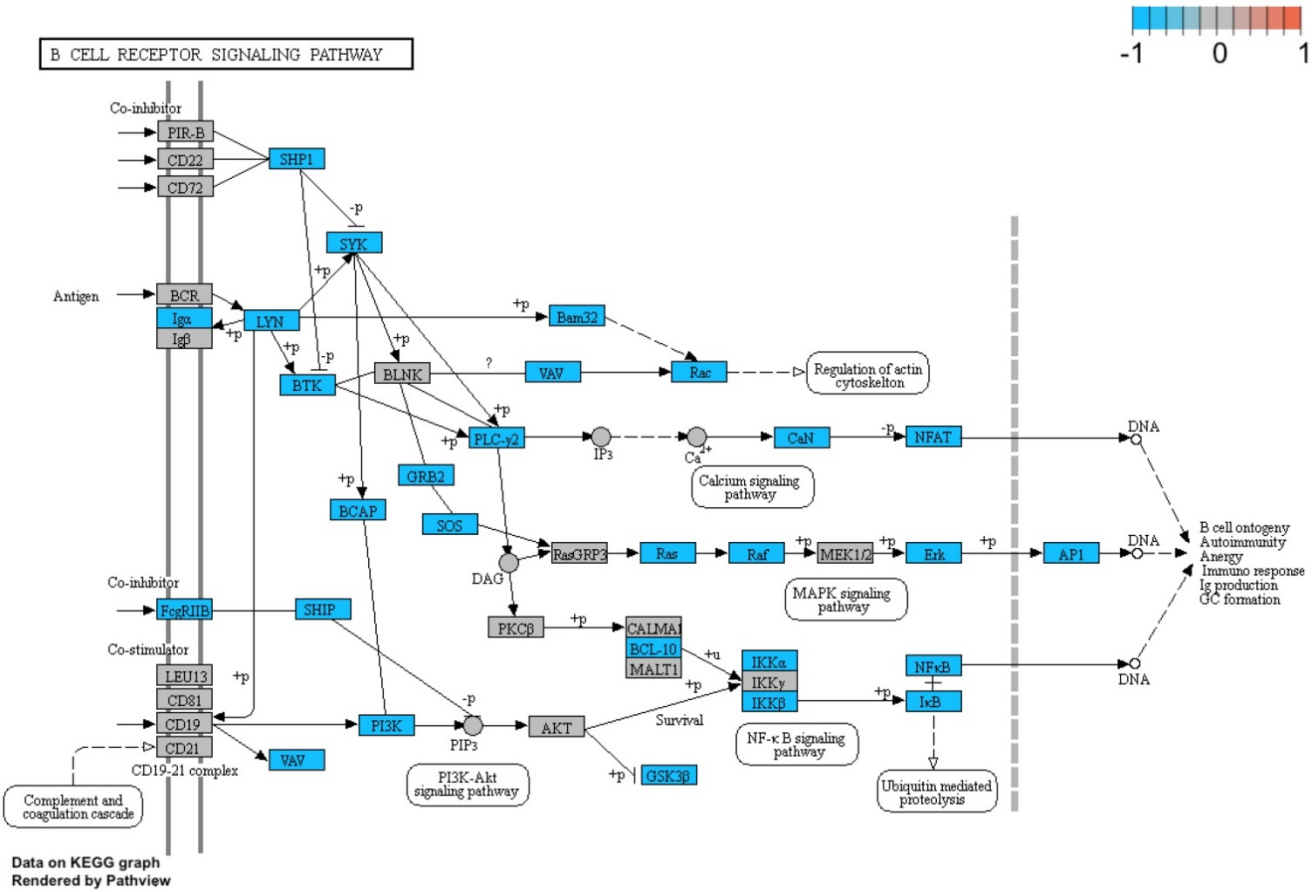
Genes and processes involved in the 04662.B cell receptor signalling pathway. The colour scale corresponds to log(FC) value, where FC stands for gene expression fold change when comparing samples from preterm to term neonates.

Additionally, we performed a detailed analysis of three signalling pathways (Figure 5: 04145.Phagosome; Figure 6: 04660.T cell receptor signalling pathway; Figure 7: 04662.B cell receptor signalling pathway), in order to better understand the effects of specific genes dysregulation on the selected process. The graphs present genes (and their up- or downregulation) and relationships between them. The majority of genes involved in these processes were significantly downregulated in samples from preterm neonates when compared to full-term infants, including genes encoding Toll-like receptors, Fc receptors, integrins, TNF-α and also genes encoding proteins involved in MAPK, NF-κB, PI3K-Akt and calcium signalling pathways.

Next, differentially expressed genes were classified according to the Gene Ontology (GO) Classification of biological processes and KEGG Database of pathways. Analysis of functional annotations mainly identified downregulated processes (e.g. signal transduction, DNA transcription, innate immune response) in PBMCs from UCB of preterm neonates when compared with term infants. The results of these analyses are displayed as separate bubble graphs for biological processes and pathways (Figure 3 and 4, respectively).

Additionally, we performed a detailed analysis of three signalling pathways (Figure 5: 04145.Phagosome; Figure 6: 04660.T cell receptor signalling pathway; Figure 7: 04662.B cell receptor signalling pathway), in order to better understand the effects of specific genes dysregulation on the selected process. The graphs present genes (and their up- or downregulation) and relationships between them. The majority of genes involved in these processes were significantly downregulated in samples from preterm neonates when compared to full-term infants, including genes encoding Toll-like receptors, Fc receptors, integrins, TNF-α and also genes encoding proteins involved in MAPK, NF-κB, PI3K-Akt and calcium signalling pathways.

**Figure 8:**
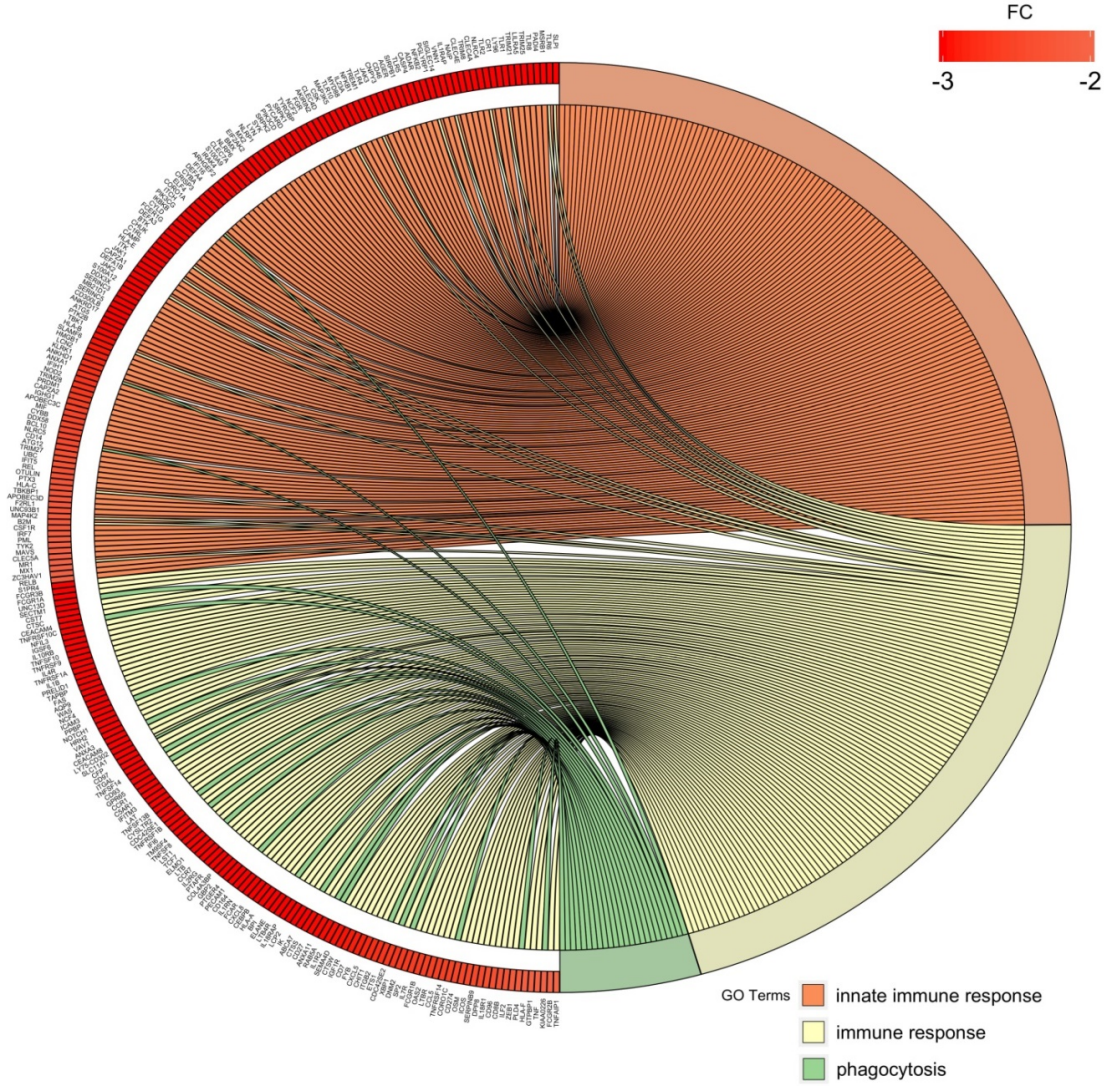
Circos plot shows the dysregulated processes (innate immune response, immune response, phagocytosis) and their associated genes in preterm neonates when compared with term infants. The level of expression for each listed gene is marked using colour scale corresponding to fold change (red = lower expression). Gene involvement in the GO terms is depicted with connecting lines and the ribbons which connect areas of the circos plots indicate also shared genes between groups.

Finally, we chose three significantly downregulated immune-related processes in preterm neonates (innate immune response, immune response, phagocytosis) and mapped the relationships between genes and GO terms in these processes with circos plots with visualization of FC (fold change) values and gene symbols (Figure 8).

Overall, the obtained results indicate significant downregulation of genes involved in immune response and related signalling pathways (e.g. phagocytosis, immunoglobulin mediated immune response, inflammatory response) in preterm infants when compared to term neonates. In particular, the expression of genes encoding several Toll-like receptors (TLRs) family members (*TLR1, 2, 4, 5, 6, 8* and *TLR10),* and of various genes associated with interleukins (e.g. *IL1RAP, IL18R1, ILF2, IL7R, IL10RB*) was decreased in PBMCs from UCB samples from preterm neonates. In addition, major signalling pathways (e.g. NF-κB, MAPK, TNF, Notch, JAK) and vital cellular processes (e.g. intracellular signal transduction, protein ubiquitination, protein transport, RNA splicing, DNA-templated transcription) were also significantly downregulated in preterm neonates in comparison with term infants.

## Discussion

Preterm birth remains the leading cause of neonatal death and both short- and long-term consequences of prematurity are detrimental. The pathogenesis of PTB remains mostly unknown, though immune-related processes have been postulated to play a significant role in it. In this study we compared the levels of complement system components and global gene expression in UCB samples between preterm and term infants. We found that levels of complement components C3a and C5a were significantly elevated in preterm babies, whereas expression of over 3,000 genes, multitude of them associated with immunity, was downregulated in those neonates in comparison with term newborns.

Foetal complement components are synthesized mainly in the liver and are well detectable around 18-20 weeks of gestation 28. Although increased complement activity in maternal circulation has been linked with PTB and elevated complement components in amniotic fluid are indicative of intrauterine infection, little or no maternal complement components are transferred to the foetus 8, 28. Our finding of elevated proinflammatory anaphylotoxins, C3a and C5a, in preterm infants is in contrary with previous studies, which in majority show that prematurity is rather related to lower levels of complement components 29, 30. Early studies from the 1970s-80s showed that the overall functional output of the classical complement pathway and the levels of the components C3, C4 and C5 were lower in preterm infants than in term neonates, whereas term neonates presented with comparatively low levels of those components in comparison with adults 29, 31. These complement deficiencies in preterm neonates are thought to be responsible for delayed inflammatory responses and impaired bacterial defences, leading to increased susceptibility to infections 32, 33. However, considerable interindividual variability in complement components concentration in neonates has been observed 34. On the other hand, the increase in anaphylotoxins in our preterm group could be attributed to the crosstalk between coagulation and complement cascades. Certain coagulation system components, such as thrombin and factor Xa, can activate C3 and C5 independently of the established complement activation pathways and these anaphylotoxins may in turn intensify coagulation 18, 35. Therefore, given that prematurity is associated with various vascular events like thrombosis and ischemia, we cannot exclude that the observed increase in anaphylotoxins levels is due to intensified coagulation and thus activation of complement cascade components.

It has also been indicated that increase in plasma complement components in apparently infection-free premature neonates could be either due to subclinical infection (enhanced activation of complement) and/or lower binding potential of complement receptors 36, 37. We cannot exclude that there were subjects with ongoing infection in our preterm group at very subclinical stage as concentrations of both CRP and IL-6 were not significantly changed in examined groups, nor can we reject the hypothesis of diminished receptor binding potential since complement component 5a receptor 1 gene (*C5AR1*) expression was significantly downregulated in our preterm neonates group (fold=-4.32), when compared to term neonates. Interestingly, several studies indicate that C3a could also have an anti-inflammatory and a neuroprotective effect in the brain tissue against neonatal hypoxia-ischemia injury 38, 39. It could indicate that in our preterm neonates group increased C3 levels might serve as a support to immature brain, rather than infection-response signal, but that remains to be further elucidated. In 1997, Wolach et al. once again confirmed lower levels of complement components in preterm neonates and added that C8 and C9 were two complement components that were the most markedly reduced at all gestational ages 40. Our study indicates low levels of C9 in preterm neonates in comparison with term babies. The C9 protein is an essential part of membrane attack complex (MAC), which induces pore formation on a cell membrane and thus causes cell lysis (e.g. bacterial lysis) 41. The C9 deficiency in neonatal serum has been associated with poor defences against bacterial pathogens 33, with premature infants having particularly low C9 levels at even higher risk of bacterial infection 42. Our results of positive correlation between complement components and gestational age suggest gradual development of this system and are in line with previous studies which show that concentrations of complement components gradually increase after birth and reach adult values in 6-18 months of age 43. Nevertheless, the dysregulation of complement in early postnatal days puts neonates at high risk of infections. This might be even more dangerous for preterm babies that are often treated with more invasive procedures that disrupt immunity barriers.

Several other studies focused on gene expression changes in maternal whole blood, or in amniotic fluid supernatant 44, 45. To date, only scarce reports on proteomic and transcriptomic profile of UCB from preterm and term neonates exist. In a recent study, Vora et al. described in UCB samples from preterm babies higher expression of genes involved in cell cycle, which, as authors suggest, might indicate focus on growth and development 46. Our study provided rather contrary results, as we observed downregulation in genes involved in cell cycle, cell division, signal transduction and DNA-templated transcription in preterm neonates in comparison with term babies. This might be attributed to different ethnic groups tested, as in our study all neonates were Caucasian, whereas in the study by Vora et al. both African American and non-Hispanic Whites infants were included. Nevertheless, our results regarding downregulation of genes involved in immune/inflammatory signalling function in preterm infants compared with term infants are in concordance with previously discussed study by Vora et al. and other reports 47, 48. In a recent meta-analysis, when eight various foeto-maternal tissue types were analysed separately, only UCB showed significant differentially expressed genes and those UCB samples that came from PTB showed downregulation in several innate immune-related pathways when compared with term samples 47. Interestingly, when the team compared dysregulated genes from UCB analysis with gene expression data from maternal blood meta-analysis, they found 13 genes including toll-like receptor 5 (*TLR5*) and other transcripts with proven involvement in immune-related processes, which were overlapping and significant in both analyses. The expression pattern of these genes involved in immune response was opposite in UCB samples (downregulation) and maternal whole blood (upregulation) in PTB group when compared with full-term infants.

Toll-like receptors (TLRs) are highly conserved evolutionary receptors of the innate immunity, which constitute the first barrier against pathogens 49-51. In our study we found significantly lower expression of not only *TLR5*, which confirms previous studies, but also *TLR1, 2, 4, 6, 8* and *TLR10* were downregulated in our preterm group, when compared with term babies. TLRs have been shown to trigger pro-inflammatory and pro-labour mediators release in uterine epithelial cells, foetal membranes and placenta, which could lead to preterm birth 52. Non-functional protein that is encoded due to nucleotide variants in *TLR5*, associated with development of bronchopulmonary dysplasia in preterm neonates 53, has also been linked with deficient immune response to flagellated bacteria 47. One of the most downregulated genes in our study, *FFAR2*, also plays a significant role in immune response to bacteria and specifically gut microbes-host crosstalk, as Ffar2 signalling modulates gut inflammatory tone and pathogen defence 54. In our study we also observed sets of genes tightly related to pathogen-stimulated response to be evidently downregulated, including genes involved in positive regulation of phagocytosis and Fc-gamma receptor signalling pathway involved in phagocytosis. The data so far on impairments in phagocytosis related to prematurity are rather inconsistent 14, 55. Recently, Posser et al. reported that phagocytosis in preterm infants is not deficient but rather preterm neonates have fewer phagocytes than term babies 56, which could be a probable factor that contributes to their vulnerability to bacterial infection.

Our results implicate that not only genes involved in innate immunity are highly downregulated in preterm infants, but also expression of those related to the adaptive immunity is lower than in term neonates. Moreover, among those under-expressed genes in our preterm group are also well-known players in multiple biological processes: JAK kinases, NOD2, MAPK kinases family, TLRs, NF-κB family and other, which may suggest inefficiencies in basic cellular processes. The deficiencies in adaptive immunity are rather understandable since adaptive immunity requires acquisition of immunological memory. Previous reports indicate that when compared to adults, neonates, and those born prematurely even more prominently, have generally lower absolute numbers of circulating lymphocytes, deficient T cell function because of more naïve T cells and less memory T cells, bias towards Th2 CD4+ T cell phenotype, reduced production of cytokines such as IFN-γ, TNF-α, IL-12 and lower production IgG and IgA antibodies 57, 58. It has been suggested that during early days of life significant changes in cell composition and gene expression occur and presumably during this extrauterine 'adaptation time' preterm babies catch up with full-term neonates 59. However, apparently before that happens they are particularly vulnerable, especially due to their deficient immune defense.

### Study limitations

This study provides interesting findings, but some limitations are present. The main limitation is unequally sized groups, which occurred due to difficulties in recruiting preterm neonates to the study. This is quite understandable though, as parents of premature infants are more reluctant to agree on their neonate participation than parents of full-term babies. Additionally, in this study women were not tested for antiphosholipid antibodies (positivity is associated with preterm birth), as in Poland this testing is recommended only in recurrent miscarriages. Another possible limitation of this study is the borderline significance of difference in IL-6 levels between tested groups, which could reflect non-clinically evident infections.

## Conclusions

Prematurely born neonates are at high risk of immediate and long-term complications following preterm birth. Disturbances in immune functions have been suggested to contribute to both preterm birth and prematurity-related complications. Our results indicate differences in complement components concentration and a significant downregulation of over 3,000 genes in UCB from preterm infants when compared with term babies. These differentially expressed genes are involved mainly in various immune-related pathways, including innate immune response, phagocytosis and TLR function. Further functional studies are required to elucidate how this gene downregulation affects pathways that they are involved in. Our findings emphasize the need for further studies on large cohorts for better understanding of postnatal stages in development of immune system.

## Figures and Tables

**Table 1:** Characteristics of the study groups. In bold, p-value<0.05 considered statistically significant.

Parameter	Preterm infants				Term infants				
Number of subjects	27				52				
Sex (M/F)	15/12				32/20				
**Parameter**	**Mean±SD**	**Median**	**Q1**	**Q3**	**Mean±SD**	**Median**	**Q1**	**Q3**	**p-value**
Gestational age [weeks]	33.0±2.6	38.5	38	39	38.4±1.0	33	32	35	**<0.001**
Weight [g]	2114.1±648.7	2010	1700	2700	3171.0±520.7	3220	2770	3500	**<0.001**
Length [cm]	46.0±5.7	46	42	50	53.5±3.5	54	51	56	**<0.001**
Chest circumference [cm]	27.4±3.3	27	26	30	32.6±2.3	33	31	34	**<0.001**
Head circumference [cm]	29.9±2.7	30	28	32	34.1±1.8	34	33	35	**<0.001**
Apgar score at 1 min	8.2±1.8	8	8	10	9.3±1.0	10	9	10	**0.001**
Apgar score at 5 min	8.9±1.2	9	8	10	9.7±0.6	10	9	10	**<0.001**
pO_2_ [mmHg]	25.2±12.6	21.8	17.6	27	19.4±13.7	14.15	12.4	20.6	**0.002**
pCO_2_ [mmHg]	46.1±6.7	44.3	41.3	50.5	50.0±8.1	49.95	45.7	53.3	**0.02**
pH	7.32±0.07	7.32	7.28	7.35	7.32±0.05	7.32	7.29	7.34	0.85
Haemoglobin [g/dL]	16.0±1.7	16.2	14.6	17.2	16.8±3.7	17.3	14.9	19.3	0.06
RBC count [10^6^/µL]	4.3±0.5	4.3	4.0	4.7	4.9±0.8	4.8	4.4	5.3	**0.001**
Platelets [10^3^/µL]	268.3±62.0	269	239	294	226.5±68.0	237	198	275	**0.01**
WBC count [10^3^/µL]	12.5±5.1	12	16.8	8.7	19.8±7.8	20	15	23.8	**<0.001**
CRP [mg/dL]	1.7±1.8	1	1	1	2.0±2.4	1	0.8	2.6	0.92
IL-6 [pg/mL]	109.3±184.0	29	11	58	19.1±17.6	16.8	3.5	21	0.07

**Table 2:** The list of 10 most upregulated genes in PBMCs isolated from UCB samples from preterm neonates in comparison with term infants.

Gene symbol	Gene name	Entrez Gene ID	Fold change
* RUNDC3A*	RUN domain containing 3A	10900	18.73
* SPTA1*	spectrin, alpha, erythrocytic 1	6708	17.98
* HLA-DRB5*	major histocompatibility complex, class II, DR beta 5	3127	17.24
* DDX3Y*	DEAD (Asp-Glu-Ala-Asp) box helicase 3, Y-linked	8653	13.42
* FHDC1*	FH2 domain containing 1	85462	12.73
* SLC4A1*	solute carrier family 4 (anion exchanger), member 1 (Diego blood group)	6521	10.35
* HLA-DRB1*	major histocompatibility complex, class II, DR beta 1	3123	9.97
* EPB42*	erythrocyte membrane protein band 4.2	2038	9.42
* SLC2A1*	solute carrier family 2 (facilitated glucose transporter), member 1	6513	8.46
* HBG1*	hemoglobin, gamma A	3047	7.59

**Table 3:** The list of 10 most downregulated genes in PBMCs isolated from UCB samples from preterm neonates in comparison with term infants.

Gene symbol	Gene name	Entrez Gene ID	Fold change
*CYP4F3*	cytochrome P450, family 4, subfamily F, polypeptide 3	4051	-14.55
*CEP19*	centrosomal protein 19kDa	84984	-14.18
*FFAR2*	free fatty acid receptor 2	2867	-13.86
*NUAK2*	NUAK family, SNF1-like kinase, 2	81788	-12.83
*TRBV20-1*	T cell receptor beta variable 20-1	---	-12.50
*FRAT2*	frequently rearranged in advanced T-cell lymphomas 2	23401	-12.45
*TNFAIP6*	tumor necrosis factor, alpha-induced protein 6	7130	-12.18
*SNORD84*	small nucleolar RNA, C/D box 84	692199	-11.99
*PI3*	peptidase inhibitor 3, skin-derived	5266	-11.43
*S1PR4*	sphingosine-1-phosphate receptor 4	8698	-11.25
